# Periampullary localized pancreatic intraepithelial neoplasia-3 (PanIN-3): evaluation with contrast-enhanced MR cholangiography (MRCP)

**DOI:** 10.2478/v10019-011-0035-2

**Published:** 2011-11-16

**Authors:** Oktay Algin, Evrim Ozmen, Pamir Eren Ersoy, Mustafa Karaoglanoglu

**Affiliations:** 1 Department of Radiology, Atatürk Training and Research Hospital, Bilkent, Ankara, Turkey; 2 Department of General Surgery, Atatürk Training and Research Hospital, Bilkent, Ankara, Turkey

**Keywords:** multidetector computed tomography, pancreatic cysts, magnetic resonance cholangiography, carcinoma, pancreatic intraepithelial neoplasia, magnetic resonance imaging

## Abstract

**Background:**

The early determination of premalignant lesions of pancreas can prevent unnecessary excessive surgical procedures and can reduce morbidity and mortality. Pancreatic intraepithelial neoplasia-3 (PanIN-3) is a preinvasive form of adenocarcinoma (carcinoma *in situ*). PanINs have not taken place in the literature of radiology yet, it should be considered in differential diagnosis of pancreatic cystic lesions.

**Case report:**

A patient with preliminary diagnosis of chronic cholecystitis who had choledocolithiasis and periampullary pancreatic cyst detected by noncontrast-enhanced (NCE) and contrast-enhanced (CE) magnetic resonance cholangiography (MRCP) is presented. Pathological examination results of gallbladder and pancreatic cyst were reported as gallbladder adenocarcinoma and PanIN-3, respectively.

**Conclusions:**

Pancreatic cystic lesions with thin septa which enhances slightly with the administration of contrast material may represent PanIN-3. In patients with cystic pancreatic lesion localized at periampullary region, using CE-MRCP together with NCE-MRCP could be useful in the evaluation of pancreatic cystic masses as well as other abdominal pathologies.

## Introduction

Pancreatic cystic lesions are often detected with imaging techniques incidentally and can be differentiated from other lesions with some characteristic imaging findings. These findings could be useful for optimal classification, accurate clinical approach, and early diagnosis and correct therapy planning. An early determination of premalignant lesions can prevent unnecessary excessive surgical procedures and can reduce morbidity and mortality. It may increase the survival of patients by providing simple surgical procedures as well.^1^

Most of pancreatic cystic lesions are pseudocyts.^2^ Serous micro-cystic adenomas, mucinous cystic neoplasms, intraductal papillary mucinous neoplasms and solid pseudopapillary tumours consist about 90% of all pancreatic tumors.^1^ The remaining 10% is made by metastases, cystic endocrine tumours, teratomas, lymphangiomas, primary pancreatic adenocarcinomas and acinar cell cystadenomas-carcinomas, etc.^1^–^3^ Although there are some reports related to the diagnostic features of the diseases mentioned above, as far we are aware, there is no paper about pancreatic intraepithelial neoplasia-3 (PanIN-3) which is a premalignant lesion (carcinoma *in situ*) in radiology literature. In this paper, we present a patient with preliminary diagnosis of chronic cholecystitis who had choledocolithiasis and periampullary pancreatic cystic lesion detected by noncontrast-enhanced (NCE) and contrast-enhanced (CE) magnetic resonance cholangiography (MRCP) examinations. Pathological examination results of gallbladder and pancreatic cyst were reported as gallbladder adenocarcinoma and PanIN-3, respectively. In this case report we aimed to discuss the role of NCE-MRCP and CE-MRCP in cystic tumours of periampullary region, to evaluate radiologic features of PanINs and review the literature.

## Case report

**FIGURE 1:**
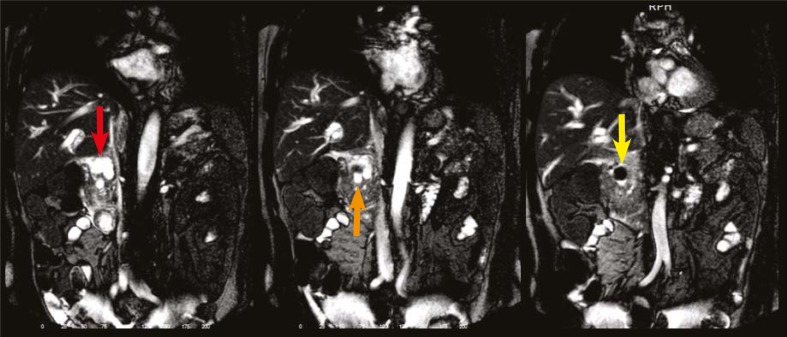
Sequential T2 weighted (noncontrast-enhanced) MR cholangiography images of the patient. The images show stone (yellow arrow) in ductus choledochus (orange arrow) and cystic lesion (red arrow) in the pancreas.

**FIGURE 2:**
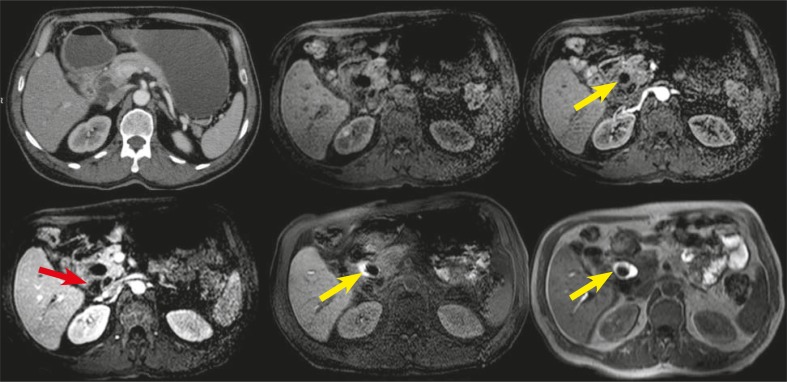
Axial contrast enhanced multidetector computed tomography (upper left), noncontrast-enhanced T1 weighted (upper medium), arterial phase contrast-enhanced T1 weighted (upper right), portal phase contrast-enhanced T1 weighted (left below), delayed phase contrast-enhanced T1 weighted with and without fat saturation (middle below and right below, respectively) images of the patient. Pancreatic cystic lesion with contrast-enhanced thin septi-wall (red arrows) can be differentiated from ductus choledochus (yellow arrows) by gadoxetic-acid enhanced T1 weighted images. Also, delayed phase T1 weighted images show stone in the ductus choledochus.

A 58-year-old man was admitted to the emergency department with right upper quadrant pain and jaundice. Though gallbladder could not be demonstrated optimally, the ultrasonographic examination revealed an increase in gallbladder wall thickness but it was contracted. Dilatation of intra and extrahepatic bile duct was detected. As a result, 64-detector multidetector computed tomography (MDCT) was performed since we could not evaluate gallbladder and choledochus optimally with ultrasound. In MDCT, gallbladder was contracted and could not be demonstrated clearly. The enlargement of choledochus and multiple choledochus stones were determined. For a better evaluation of the biliary stones, T2 weighted (T2W) MRCP (NCE-MRCP) was performed. T2W images showed multiple stones in ductus choledochus and intrahepatic bile ducts. A 15×20 mm cystic lesion including thin septa was demonstrated at the head of pancreas at periampullary region (Figure 1). There was no relationship between cystic lesion and pancreatic duct. Gadoxetic-acid enhanced MRCP (CE-MRCP) was performed at the same session to examine the bile duct obstruction and to evaluate the pancreatic cystic lesion (Figure 2). A slight enhancement was detected at the wall and septi of the cystic lesion in CE-MRCP. There was no relationship between cyst and choledochus or pancreatic duct. There was no obstruction in the biliary tract as well. The transition of contrast material to the lumen of gallbladder was not seen at either early or late phase images. The patient was planned to undergo the surgical treatment according to these findings.

At surgical exploration, the size of gallbladder was significantly reduced. There were fibrotic adhesions between gallbladder, liver and transverse colon. Cholecystectomy was done; the pancreatic cystic lesion was aspirated and excised, as well. The histopathologic examination revealed a moderately differentiated adenocarcinoma of gallbladder with positive surgical margins and the pancreatic lesion was reported as PanIN-3. The patient was referred to the medical oncology department. The patient was in good condition the 6^th^ month after the operation.

## Discussion

The most important features of pancreatic adenocarcinomas are their progressive course and their high mortality rate. The majority of patients have locally advanced or distant metastatic disease (inoperable stage) during the diagnosis. Therefore, a detection of premalignant and malignant pancreatic lesions at early stage is necessary for curative surgery and improving the survival rates like in other oncological diseases.^4^–^6^

PanIN is the most common and histologically well-defined precursor that leads to pancreatic ductal carcinoma.^7^ Histologically PanINs could be divided into 3 subtypes. PanIN-1 and PanIN-2 are low-grade lesions, whereas PanIN-3 (carcinoma *in situ*) is a preinvasive form of adenocarcinoma.^7^ In conclusion PanIN-3 is a premalignant lesion similar to intraductal papillary mucinous neoplasm and mucinous cystic neoplasm and these lesions should be diagnosed early before they improve to invasive carcinoma. Although PanINs have not taken place in the literature of radiology yet, it should be considered in differential diagnosis of pancreatic cystic lesions.^3^ The existence of genetic, biochemical, and histological relation between PanINs and hepatobiliary/pancreatic carcinomas was reported in recent pathology literature.^4^,^7^,^8^ Gallbladder adenocarcinoma of our patient is a good example for this situation.

To evaluate the relation between the lesion and pancreatic duct at the heavily T2W sequences (NCE-MRCP) may differentiate PanIN-3 from intraductal papillary mucinous neoplasm.^2^ Although NCE-MRCP cannot always show the relationship, it often contributes to the diagnosis.^3^ Endoscopic ultrasound and endoscopic ultrasound-guided fine needle aspiration might be useful when NCE-MRCP is inadequate.^3^ Mucinous cystic neoplasms are often seen among women during the 4–6^th^ decades (also called mother lesions).^1^ They consist of one or multiple cystic lesions and 10–25 % of the lesions may include peripheral curvilinear calcification. They are well demarcated and generally larger than 2 cm.^1^ Wall or septa of cysts could be enhanced after the contrast material administration similar to PanIN-3.^2^

Serous cystadenomas are generally seen among women after the 6^th^ decade (also called grandmother lesions).^1^ They include fibrous central scars with or without a characteristic stellate pattern of calcification and these fibrous central scars are enhanced with contrast material.^1^ Demonstration of fibrous central scars with or without other characteristic features could provide differentiation serous cystadenomas from other cystic lesions radiologically. Pseudocysts should be included to the differential diagnosis of pancreatic cystic lesions. Also, percutaneous or endoscopic ultrasound-guided fine needle aspiration of cystic fluid may be helpful in many cases with pancreatic cystic mass; since levels of amylase increase in the cystic fluid of pseudocysts, CEA levels are elevated in many mucinous cystic neoplasms, keratinous and amorphous debris can be seen in lymphoepithelial cysts, and mucin-rich fluid and columnar mucinous cells within this fluid can be observed in intraductal papillary mucinous neoplasm.^1^–^3^ Solid pseudopapillary tumour and acinar cell cystadenoma/carcinoma are the other rare cystic neoplasms that should be considered in differential diagnosis as well.^1^,^2^,^9^

MDCT, abdominal magnetic resonance imaging (MRI) and MRCP were reported as most useful techniques to evaluate pancreatic cystic lesions.^3^,^10^ Nevertheless, periampullary cystic lesions cannot be evaluated optimally by the tomographic examination in patients with dilated distal choledochus and choledocholithiasis as in our case.^11^ In such cases MRI and NCE-MRCP are supposed to be better in determination of cyst morphology and classification.^11^ However, there is not a published report related to the role of CE-MRCP in the evaluation of pancreatic cysts. CE-MRCP could be useful in determination of the relationship between the cystic lesions adjacent to choledochus and pancreatic duct or choledochus itself. It may also detect possible causes of the pancreatic duct obstruction at the periampullary region and it can provide functional data. CE-MRCP could evaluate the entire abdomen (especially in hepatobiliary system, duodenum, and pancreatic evaluation) at a single session with the addition of T2W images (NCE-MRCP).^12^ Moreover, CE-MRCP could supply an optimal assessment in case with suspicious malignant cyst by the detection of the contrast enhancement of cystic lesion with dynamic sequences, without a risk for radiation exposure or nefrotoxic contrast-material application.

## Conclusions

Pancreatic cystic lesions with thin septa which enhances slightly with the administration of contrast material may represent PanIN-3. Early diagnosis and treatment of these lesions increase the duration and quality of lifetime by preventing excessive surgical procedures. Therefore, radiologists should be aware of the premalignant pancreatic cystic lesions. In patients with cystic pancreatic tumour localized at the periampullary region, using CE-MRCP together with NCE-MRCP could be useful in the evaluation of pancreatic cystic masses as well as other abdominal pathologies.
